# Dental Education

**Published:** 1883-12

**Authors:** 


### Editorial.

### DENTAL EDUCATION.

It is a hopeful sign of the times when so much of the attention of the profession is directed toward the proper preparation and training of those who are to fill the dental ranks when the men now in active practice shall have passed away. The growth of modern dentistry has been wonderfully rapid, and the supply of educated men has not been equal to the demand. With the introduction of vulcanite as a base for artificial teeth, and the consequent cheapening of dentures, there came a demand for dental services that was undreamed of by the old gold and silver workers. The manipulation of the new base was simple and easily learned, and forthwith there sprang into practice a great cloud of cheap dentists, whose professional knowledge was limited by the ability to extract teeth and insert rubber plates. They were usually men of little general education, and of no personal ambition. There was no such thing as dental legislation, and any one was quite at liberty to put up his sign of "surgeon dentist," and seize such prey as came within his reach. He needed no instruments or tools, save a few pairs of forceps and a vulcanizer, and so very little capital was demanded, and soon hundreds of these charlatans and ignorant pretenders were traveling the country over, extracting teeth by the wholesale, and putting in the merest burlesques upon dentistry in the shape of rubber dentures. It did not take very many years of such practice to teach people that this was not dentistry, and public sentiment soon sanctioned the passage of laws so regulating dental practice as to limit or prohibit this irruption of quacks.

The intelligent and educated honorable men in the profession are to-day working to secure a better class of students, and to bring into dentistry practitioners who may be a credit to their calling, or who at least shall not positively disgrace it. But the Almighty Dollar has charms for many who pass for representative men among us; charms which entirely outweigh their professional pride, their regard for their professional brethren, their appreciation of the responsible position which some of them are called to fill, and even their character for professional honesty. While individual dentists, many of them, are carefully scrutinizing the qualifications of such students as apply to them to act as their preceptor, there are professors in colleges, deans and secretaries, who are assisting to weigh down a profession which has honored them, by periodically grinding out batches of graduates who are little better qualified to undertake a reputable practice than were the self-taught rubber workers who rushed into dentistry fifteen or twenty years ago. It is a fact which causes reputable dentists to blush with mortification and shame, that some of our colleges are little better than mere diploma mills. They rush students through a course of instruction that is ridiculously insufficient. They seize upon the flimsiest pretexts for shortening the term of pupilage. They matriculate boys who have not sufficient education to be able to comprehend the lectures to which they are supposed to listen. They welcome students with not enough of intelligence to learn to properly make a horse-shoe, and at the very shortest notice turn them out fully equipped professional men, armed with a diploma of the true signification of which they have not the faintest conception. There is no graded course, but the plough-boy who has not decently mastered the commonest elements of learning graduates as quickly as though he had been a student all his life. But one qualification seems to be required, and that is the ability to pay the fees.

We are not making an indiscriminate charge against all our colleges, for the most of them are, we believe, more conscientious in the administration of the powers granted them. But there are those, and they pretend to a respectable standing among colleges too, which seem to be entirely dominated by a contemptibly sordid, money-grubbing spirit. It is time that a halt was called, and an account demanded by the profession of the way in which some of these men are administering the trusts reposed in them. Common self-respect will soon force us to this, for the D. D. S. is a distinctively American degree, and we cannot expect the people of other countries to hold in good repute a diploma that is so abused.

It is in the heat of a righteous professional indignation, aroused by the perusal of the following letter received by us from an undergraduate in one of our schools, that we write these things. The man who penned this beautiful exhibition of scholarship is a pupil in a dental college, and another year will see him launched upon a suffering community, a full fledged D. D. S., armed with a diploma bearing the signatures of men of high standing in dentistry, who attest his eminent fitness for professional honors. We print the letter exactly as it was received, merely suppressing name and locality, though we are strongly tempted to give the college at which he is a matriculant a little free advertising.

*Mr. Barrett, Esq.*

Dear Sir I was informed by one of the Dental Depots the other day about you as a Dentist which I am here with the —— Dental College taking a course which make me in the same line with you also and if possible I would after this term like to come and work with or for you after this term as I will not or cannot get through this year so I will have to be Idle all summer if I do not suceede in geting in some plase and would like to come and practice with you this spring untill next fall which I would be very much pleased to do and hope you have a plase open for me. Please write soon.

I remain yours Resp., (—— —— ——.)

Care of the —— Dental College.

